# Commentary: Why Don't You Go to Bed on Time? A Daily Diary Study on the Relationships Between Chronotype, Self-Control Resources and the Phenomenon of Bedtime Procrastination

**DOI:** 10.3389/fpsyg.2018.00915

**Published:** 2018-06-11

**Authors:** Floor M. Kroese, Marieke A. Adriaanse, Catharine Evers, Joel Anderson, Denise de Ridder

**Affiliations:** ^1^Department of Social, Health and Organizational Psychology, Utrecht University, Utrecht, Netherlands; ^2^Department of Philosophy and Religious Studies, Utrecht University, Utrecht, Netherlands

**Keywords:** bedtime procrastination, self-regulation, sleep, health behavior, commentary

Kühnel et al. (2018) challenge our viewpoint (Kroese et al., 2014, 2016a; as well as Kroese et al., 2016b; Kamphorst et al., 2018) that bedtime procrastination—going to bed later than intended without having external reasons for doing so—is a self-regulation problem. They argue that bedtime procrastination is *not* a self-regulation problem because it is predicted by chronotypes. Below we outline why we disagree and how framing the issue of bedtime procrastination as a self-regulation problem is explanatorily relevant, theoretically sound and practically fruitful, allowing for a more constructive approach to this issue, including the development of interventions.

First and foremost, it is important to point out that, contrary to what Kühnel et al. imply, pointing to a “mismatch between [biological drives] and societal requirements” does *not* demonstrate self-regulation to be irrelevant to the explanation of a phenomenon. On the contrary, such mismatches often lie at the very basis of self-regulation issues. Consider for example eating behavior as a typical self-regulatory challenge: eating behavior often involves a conflict between the biological drive to eat sugary, fatty foods vs. the (society-imposed and/or personal) desire to maintain a slim figure. This does not mean that eating behavior, or other behaviors “resulting from an interplay of genetic influences and environmental factors” do not involve self-regulation, as the authors suggest (p. 2). The role of self-regulation is in fact essential, as the extent to which people are able to manage such conflicts between biological predispositions and societal requirements (i.e., to self-regulate) affects the degree to which each factor determines behavioral outcomes (see also Kotabe and Hofmann, 2015).

Biological drives do, of course, influence the *magnitude* of self-regulation conflicts. We would concur with Kühnel and colleagues that it is particularly difficult for people with late chronotypes to go to bed at the times that are required by the work environment of their study's participants, just as maintaining a healthy diet is more challenging for people with a predisposition to favor sweet or fatty foods (Bouchard, 2007). That is, given typical social expectations regarding wake-up times, people with late chronotypes will experience greater conflict and thus need more self-regulation than people with early chronotypes. Therefore, their hypothesis that bedtime procrastination should occur more often among later chronotypes is completely in line with our own self-regulation perspective and does not—as the authors imply—challenge it. Specifically, we would predict an interaction between chronotype and trait self-control such that bedtime procrastination is most likely to occur among people with late chronotypes and low trait self-control.

The data reported in the paper demonstrated that the relation between chronotype and bedtime procrastination was moderated by time of the week, such that chronotype was only related to bedtime procrastination on days earlier in the work week compared to days later in the work week. The authors imply that this finding is in contradiction with the notion of bedtime procrastination as a self-regulation issue. However, their biological explanation (i.e., that accumulated sleep debt over the course of the week increases the homeostatic sleep drive, which counteracts the circadian drive for arousal) again does not preclude the self-regulation perspective. Indeed, we would say that if the *conflict* between the drive to stay up late and the need to get sufficient sleep is reduced over the course of the week—either because people's biologically preferred bedtimes become earlier due to accumulated sleep debt, or because the need to get up early is reduced (in the weekends)—the role of self-regulation is rendered less important.

Finally, a reply is in order regarding perhaps the most striking finding of Kühnel et al's study, namely, that “on evenings on which employees indicated to have *more* self-regulatory resources at their disposal…employees showed *more* bedtime procrastination.” To begin with, it should be noted that a recent study suggests the opposite (negative) correlation (Kamphorst et al., 2018). The point we wish to emphasize here, however, especially in light of ongoing disputes about resource-based approaches to self-regulation (e.g., Inzlicht and Berkman, 2015), is that the self-regulation perspective on bedtime procrastination need not rely on an explanation in terms of the momentary self-regulation resources on which Kühnel et al. focus. Even if bedtime procrastination were poorly explained by resource-based approaches, self-regulation, understood as a *trait*, could still play a central role in bedtime procrastination. Rather than relying on willpower to turn their intentions into actions, successful self-regulators are known to make use of adaptive effortless strategies (e.g., habits) that are not affected by variable mindsets or states (Gillebaart and de Ridder, 2015). For example, it is plausible that better self-regulators have better sleep hygiene habits (e.g., dimming the lights in the evening) that make it easier for them to go to bed on time, regardless of their temporary resources. Our hypothesis, which would need to be tested experimentally, is that supporting such self-control strategies in people with late chronotypes can reduce bedtime procrastination.

In sum, we believe that reducing bedtime procrastination to a predominantly biological issue does not do justice to its complexity and, more importantly, precludes the development of interventions for which the self-regulation perspective can provide innovative and practical starting points. That is, while Kühnel et al. have made an important contribution by identifying late chronotypes as a risk factor to bedtime procrastination, we firmly oppose reducing the complex phenomenon of bedtime procrastination to a biological issue. Doing so would preclude the identification of behavioral patterns contributing to going to bed late as well as the development of interventions seeking to alter these behavioral patterns. Thus, we particularly advocate research integrating findings from biological, psychological, and social perspectives to advance our knowledge on the topic of bedtime procrastination, as it is undisputed that getting insufficient sleep by going to bed late can have negative consequences.

## Author contributions

FK drafted an initial version of this commentary, MA, CE, JA, and DdR provided substantial comments and suggestions for improvement.

### Conflict of interest statement

The authors declare that the research was conducted in the absence of any commercial or financial relationships that could be construed as a potential conflict of interest.
